# Undergraduate Students’ Social Entrepreneurial Intention: The Role of Individual Environmental Responsibility and Absorptive Capacity

**DOI:** 10.3389/fpsyg.2022.829319

**Published:** 2022-04-14

**Authors:** Cheng-Min Chao, Tai-Kuei Yu

**Affiliations:** ^1^Department of Business Administration, National Taichung University of Science and Technology, Taichung, Taiwan; ^2^Department of Business Administration, National Quemoy University, Kinmen, Taiwan

**Keywords:** social capital, individual environmental responsibility, individual absorptive capability, social entrepreneurial intention, formative model

## Abstract

As social entrepreneurial intention has received increasing attention from scholars and practitioners, no clear conclusions have been drawn regarding antecedent or external factors that influence social entrepreneurial intention. This study aims to develop a structural model to shape the social entrepreneurial intention of business administration students, which involves the theory of planned behavior (i.e., attitudes, subjective norms), social capital, individual environmental responsibility, and individual absorptive capacity (i.e., potential absorptive capacity and realized absorptive capacity). Furthermore, this study regards potential absorptive capability as a multi-dimensional construct of a higher-order structure. The participants were students from business administration colleges/universities (including general universities and science and technology universities) in Taiwan. The empirical data from 969 participants were analyzed using Smart PLS 3.0 to obtain the results. The results revealed that: (1) social capital had a significant positive effect on attitudes and subjective norms; (2) attitudes, subjective norms, individual environmental responsibility, and realized absorptive capability had a positive effect on social entrepreneurial intention; however, social capital and potential absorptive capability had a negative effect. The results were discussed, and some specific recommendations for practitioners of business administration education were proposed.

## Introduction

Proposed by the United Nations (UN) in 2015, the Sustainable Development Goals (SDGs) framework has since become the focus of discussion and policy work when addressing major social challenges (Günzel-Jensen et al., 2020). Social entrepreneurship (SE) can be a society’s best healer because it pursues economic, social, and environmental goals concurrently (Nigam et al., 2014; Hoa et al., 2021). After all, SE’s ultimate goal is to solve society’s problems “effectively.” For social entrepreneurs, the goal of the SDG framework mandates that they participate and reach a consensus with local partners and then use their knowledge, adaptability, and innovation capabilities to contribute to society. Therefore, the SDG framework is also expected to bring huge benefits to SE ventures. The Internet has significantly impacted many aspects of human life and become an indispensable component of individuals’ daily activities and entrepreneurship. In line with this phenomenon, many non-profit organizations (NPOs) in Taiwan have successively developed online platforms in recent years to provide entrepreneurs with opportunities to engage in online SE, an example being the Taiwan NPO Information Platform. The ultimate goal of such endeavors is to pursue economic, social, and environmental equilibrium concurrently.

Social entrepreneurship places the focus of entrepreneurial intention (EI) on the launch and development of enterprises that solve social problems and needs (Prasetyo, 2016; Barton et al., 2018; Zaremohzzabieh et al., 2019; Sahasranamam and Nandakumar, 2020; Santos et al., 2020; Hoa et al., 2021). Scholars and practitioners have paid more attention to social entrepreneurial intention (SEI) and emphasized its importance as the first step toward SE behaviors. Over the past two decades, an increasing number of studies have determined that SE is a potential strategy for responding to social needs. However, there is a relative lack of theory and inquiry to ascertain the antecedent factors affecting SEI (Zaremohzzabieh et al., 2019), making an examination of these factors necessary (Dwivedi and Weerawardena, 2018; Sahasranamam and Nandakumar, 2020; Hoa et al., 2021). In the SEI research field, the theory of planned behavior (TPB) proposed by Ajzen (1991) is the most widely used and tested and suffices to serve as its theoretical background (Salamzadeh et al., 2013; Cavazos-Arroyo et al., 2017; Ernst, 2018; Zaremohzzabieh et al., 2019). In addition, the original constructs of the TPB model can be easily modified for application to various research fields (Salamzadeh et al., 2013; Zaremohzzabieh et al., 2019). Hence, some SEI researchers have suggested broadening the TPB structure to enhance the quality of the results and explanatory power of behavioral intentions (e.g., Salamzadeh et al., 2013; Chen and Hung, 2016; Cavazos-Arroyo et al., 2017; Ernst, 2018; Zaremohzzabieh et al., 2019).

The perspective of social capital (SC) has increasingly gained attention in management and plays a vital role in the molding of EI (Anderson et al., 2007; Rodrigo-Alarcón et al., 2018; Mahfud et al., 2020). In recent years, many contributions to entrepreneurship research were made from the SC theory and TBP. However, only a few studies have linked the two theories. For entrepreneurs preparing to start a business in dynamic environments, the keys to successful entrepreneurship and EI include the continuous acquisition, assimilation, transformation, and exploitation of new knowledge and to do so flexibly. Our understanding is that previous literature on the absorptive capacity (AC) theory focuses on managing corporate innovation and the innovation of business models (Audretsch et al., 2021; Miroshnychenko et al., 2021; Qi et al., 2021). This is usually discussed from an organizational perspective but rarely from a personal perspective. The relationship between individual absorptive capacity (IAC) and SEI has not been the subject of previous research. To address this gap in the literature, IAC was applied to the research on undergraduate students’ SE in this study. The aim was to understand the impact of IAC on their SEI.

Many researchers from various fields have devoted themselves to discussing environmental responsibility (ER), given its important role in the past few years. This has led to a rapid increase in ER research topics and extensive attention being paid to it and its related applications (Yang et al., 2021). Yang et al. (2021) emphasized the role of stakeholders participating in ER activities. They argued that although the existing literature discusses stakeholders as a factor, the participation of knowledge institutions (including colleges and education departments) and discussion from the demand-side perspective is lacking. Therefore, they proposed that attention be paid to the role of knowledge institutions in the ER field. In addition, many studies focus on the economic benefits and economic and environmental performance of ER, but relatively little attention is paid to its social performance (including entrepreneurship and job creation). In this study, the impact of individual environmental responsibility (IER) on SEI was examined to fill the void in the literature. Given the important role of entrepreneurship, the Taiwan government has emphasized implementing entrepreneurship education in higher education in recent years and developing entrepreneurship programs for undergraduate students (such as student entrepreneurship clubs and entrepreneurship training). Through the provision of such programs, the aim was to enhance the capabilities of new entrepreneurs. Despite a large amount of literature dedicated to SEI, there is limited understanding of the antecedent or external factors affecting the SEI of undergraduate students in colleges of business and management. In particular, little is known about the impact of SC, attitudes, subjective norms, IER, and IAC on SEI. Using the valence framework as the basis, the impact of SC, attitudes, subjective norms, IER, and IAC (i.e., potential absorptive capacity and realized absorptive capacity) on SEI was considered in this study.

The specific research questions of this study were (i) does SC affect attitudes and subjective norms? and (ii) do SC, attitudes, subjective norms, IER, and IAC (i.e., potential absorptive capacity and realized absorptive capacity) affect SEI? The SEI model was developed and evaluated to examine the antecedent factors affecting the SEI of undergraduate students in colleges of business and management to answer these questions. This was done from various aspects, including SC, attitudes, subjective norms, IER, and IAC (i.e., potential absorptive capacity and realized absorptive capacity). Correspondingly, the specific aims of this study were to (i) examine the formation of undergraduate students’ decisions on SEI using the TPB model and (ii) understand the impact of SC, IER, and IAC (i.e., potential absorptive capacity and realized absorptive capacity) on their SEI. The study makes two academic and practical contributions. First, a conceptual model based on the perspectives of SC, IER, and IAC was proposed to study their impact on the SEI of undergraduate students in business and management. Second, it was proposed that the potential and realized ACs of IAC are multi-dimensional constructs with higher-order structures.

## Literature Review and Hypotheses Development

### Social Entrepreneurial Intention

The rapid development of Internet has led to changes in entrepreneurship and enabled it to flourish entirely or partially in the virtual world (Barton et al., 2018; Chandna and Salimath, 2018). The new environment for online entrepreneurship differs from the traditional physical environment and allows enterprises and users to connect seamlessly in a variety of ways. The global effort in recent years has been directed toward society and the environment. A sustainable world is regarded as one of the important goals of sustainable development, and the SDG framework is proposed.

Social entrepreneurship is a dynamic process initiated by social entrepreneurs with unique capabilities, concepts, personalities, and behavioral traits. Their specific products are social enterprises, which are oriented toward social missions and aim to solve social problems or achieve social goals (Mair and Marti, 2006; Huybrechts and Nicholls, 2012; Prasetyo, 2016). Mair and Marti (2006) proposed that SE uses and combines discovered resources innovatively and makes use of opportunities fully. Its purpose is to catalyze social changes and uphold the spirit of sustainability when responding to human needs. The discussion of EI is one of the important core concepts of entrepreneurial research. It refers to an individual’s state of mind with a focused mentality, concentrating their attention and experiences on planned entrepreneurial behaviors before taking action, such as entrepreneurship and becoming an entrepreneur” (Moriano et al., 2012; Do and Dadvari, 2017). EI is directed by SE to concentrate on launching and developing enterprises that solve social problems and needs. Therefore, many studies have determined SE to be a potential strategy for responding to social needs.

### Social Capital Theory

Social capital (SC) is a combination of social resources cited by sociologists, economists, management specialists, and scientists (Yan and Guan, 2018). The SC concept is essential to the value creation process and realization of innovation (Nahapiet and Ghoshal, 1998). Coleman (1988) defined SC from the functional aspect of social structure. As a resource within that structure, it allows people to exchange information with one another by interacting and through mutual trust. Members in the social structure achieve specific expected goals in the process, thereby forming sustained social relationships. Coleman (1990) proposed that the SC relationship consists of four dimensions: (1) obligations and expectations, (2) information potential, (3) norms and effective Sanctions, and (4) authority relations. Nahapiet and Ghoshal (1998) defined SC as the sum of actual and potential resources embedded within, available in, and derived from the network relationships owned by individuals or social units (Nahapiet and Ghoshal, 1998). In other words, social interactions develop social capabilities to generate value (Mahfud et al., 2020).

Social capital is composed of three distinct dimensions: (i) the structural dimension is the organizing mode for actors to connect; (ii) the cognitive dimension is the meaning and understanding shared by all parties in the network; and (iii) the relational dimension is the type of personal relationships that people in the network have. Some researchers opine that it is important to study SC through these three dimensions (Zhao et al., 2012; Rodrigo-Alarcón et al., 2018; Yan and Guan, 2018). After its proposal, the concept of dimensions was mainly adopted by subsequent researchers when measuring SC (Zhao et al., 2012; Rodrigo-Alarcón et al., 2018; Yan and Guan, 2018). SC is one of the most extensively applied concepts in the social sciences. Although there were important impacts when the SC concept was applied in different fields, its definitions varied (Ahmed, 2018; Yan and Guan, 2018). In this study, the definition of SC proposed by Nahapiet and Ghoshal (1998) was adopted; namely, it is the value that individuals acquire by developing their social capabilities through social interactions.

According to SC, multilevel social interactions during the entrepreneurial process are highly beneficial to entrepreneurs, be they individuals or groups (Anderson et al., 2007; McKeever et al., 2014; Zaremohzzabieh et al., 2019; Mahfud et al., 2020). Malebana (2016) proposed the application of TPB and SCT to EI formation. Research shows that TPB is an important model for understanding the relationship between SC and EI. Specifically, SC is significantly related to attitudes, perceptual behavior control (PBC) and EI. Zaremohzzabieh et al. (2019) proposed the importance of SC as antecedent factors for TPB (attitude, subjective norm, PBC), and argued that SC affects attitude, subjective norm, PBC. From these arguments, this study proposed that SC affects attitudes and subjective norms. Therefore, we propose the following hypothesis:

Hypothesis 1: Social capital has a significant influence on attitude.
Hypothesis 2: Social capital has a significant influence on subjective norm.

Previous studies pointed out that SC is of great value to the formation and success of SE (Newman and Dale, 2005; Malebana, 2016; Ernst, 2018). Anderson et al. (2007) pointed out that SC is indispensable in the entrepreneurship process and also play an important role in shaping EI (Mahfud et al., 2020). Malebana (2016) found that SC affects SEI. Ernst (2018) pointed out that SC has a positive relationship with SEI. From these arguments, this study argues that SC to be an important antecedent factor in SEI and proposes the following hypothesis:

Hypothesis 3: Social capital has a significant influence on social entrepreneurial intention.

### Theory of Planned Behavior

The entrepreneurial event model (EEM) proposed by Shapero and Sokol (1982) and TPB proposed by Ajzen (1991, 2015) are the most commonly used psychological theoretical models in entrepreneurial research to predict and explain individual EI (Paul and Shrivatava, 2016; Tsai et al., 2016; Esfandiar et al., 2019). Many empirical studies (Salamzadeh et al., 2013; Paul and Shrivatava, 2016; Tsai et al., 2016; Cavazos-Arroyo et al., 2017; Ernst, 2018; Zaremohzzabieh et al., 2019) have confirmed that TPB by Ajzen (1991, 2015) is the most effective and well-developed theoretical framework for examining EI. This theory is mainly used to predict and explain individuals’ behavioral patterns under specific situations. It posits that when intentions predict behaviors, certain specific attitudes will predict and influence those intentions (Ajzen, 1991). These behavioral intentions originate from attitudes, which play a vital role in creating intentions and become the determining factors of behaviors (Salamzadeh et al., 2013; Do and Dadvari, 2017).

The results of previous empirical studies show that the TPB model seems to suffice as the theoretical background for SEI formation (Salamzadeh et al., 2013; Cavazos-Arroyo et al., 2017; Ernst, 2018; Zaremohzzabieh et al., 2019). Three constructs form the core of that model: (i) attitude, which is the degree to which a person thinks whether a particular behavior is attractive or not; (ii) subjective norms, which refer to the perceived social pressure to perform particular behaviors or not; and (iii) PBC, which is the ease or difficulty that individuals have over their behavioral performances. These three constructs increase SEI and enhance the chances of SE success (Cavazos-Arroyo et al., 2017; Ernst, 2018; Zaremohzzabieh et al., 2019). Zaremohzzabieh et al. (2019) found that attitudes, subjective norms, and PBC affect SEI. Summarizing the discussion above, this study proposed that attitudes and subjective norms increase SEI. Therefore, the following hypothesis was proposed:

Hypothesis 4: Attitude has a significant influence on social entrepreneurial intention.
Hypothesis 5: Subjective norm has a significant influence on social entrepreneurial intention.

### Individual Environmental Responsibility

The United Nations’ *Report for the 2030 Agenda for Sustainable Development* stated that developing a sustainable world is one of the important goals of sustainable development. An important way to achieve the SDGs is to encourage enterprises to undertake ER (United Nations, 2015). Early research on corporate ER regarded ER as an important part of social responsibility (Han and Cao, 2021). With people paying increasing attention to the sustainable development of the environment, ER and social responsibility have gradually become separate research topics in recent years, with the former becoming an independent research subject. Many published papers have indicated that ER is receiving widespread attention in academic research and is becoming more important in organizational practice (Peng and Mao, 2018; Wei et al., 2018; Jia et al., 2021).

Environmental responsibility refers to the responsibilities and obligations that individuals bear when dealing with environmental issues in the process of exploiting, utilizing, managing, and protecting resources (Wei et al., 2018). Jia et al. (2021) defined ER as individuals’ sense of responsibility to prevent environmental degradation or solve certain ecological problems. Peng and Mao (2018) believed that ER is embodied in individuals’ determination, courage, and spirit of dedication to solve ecological and environmental issues. More importantly, individuals who possess these characteristics form a firm sense of internal trust, which prompts them to assume ER, strengthening their IER and environmental behaviors. This study supported Peng and Mao (2018) that ER manifests in individuals’ determination to solve ecological and environmental problems. Therefore, IER was adopted for discussion in this study. Based on the concept of ER proposed by the scholars mentioned above, IER was defined as “individuals’ determination, spirit of dedication, and sense of responsibility to prevent environmental degradation and solve ecological and environmental problems.”

Although ER research has gradually received more attention lately, most studies focused on examining the impacts of corporate ER on the economic, environmental, and social performances of enterprises. Research on stakeholders’ participation in ER activities has similarly been based predominantly on enterprises (Chen et al., 2019; Han and Cao, 2021; Yang et al., 2021). In contrast, Yang et al. (2021) proposed a lack of demand-side perspective regarding the participation of knowledge institutions (including colleges and education departments) in ER activities. From a personal perspective, individuals’ internalized ER is awakened when they face a dilemma between personal and social interests. They are then prompted to implement environmental protection behaviors. Therefore, this study proposed that when undergraduate students are determined and willing to take responsibility for preventing environmental degradation and solving ecological and environmental problems, they will be motivated to implement environmental protection activities and improve social performance through SE. This led to the following hypothesis:

Hypothesis 6: Individual environmental responsibility has a significant influence on social entrepreneurial intention.

### Absorptive Capacity Theory

Innovation and entrepreneurship require additional external knowledge and information (Audretsch et al., 2021; Qi et al., 2021). The concept that is commonly related to innovation and learning is AC. On the topic of entrepreneurship, the ability to absorb externally and internally created knowledge is identified as a key source of entrepreneurial capabilities (Audretsch et al., 2021). Cohen and Levinthal (1990) first introduced the construct of AC to explain the ability of organizations to recognize, acquire, and exploit external knowledge to innovate and adapt. They conceptualized AC as enterprises’ ability to recognize the value of new information, assimilate it, and then apply this knowledge to commercial ends. Cohen and Levinthal (1990) proposed that AC is composed of cognitive, digestive, and application abilities. Based on the AC concept put forward by Cohen and Levinthal (1990), its most important result lies in innovation and innovative outputs. Their perspective was studied and applied by many subsequent researchers, which further cemented the belief that AC is the core factor for enterprises to maintain their competitive advantages (Zahra and George, 2002; Stulova and Rungi, 2017).

A re-conceptualization of the model originally proposed by Cohen and Levinthal (1990) was made from the perspective of dynamic capabilities by Zahra and George (2002). They defined AC as the ability of enterprises to acquire, assimilate, transform, and exploit knowledge before setting the organizational norms and processes that enable them to produce dynamic capabilities. They redefined AC as two distinct but related capabilities, namely, potential and practical ACs. Potential AC consists of the acquisition components (enterprises’ ability to analyze, explain, and understand external knowledge) and assimilation components (enterprises’ ability to analyze, explain, and understand external knowledge). Practical AC refers to enterprises’ transformation and exploitation of external knowledge or their utilization of knowledge. This consists of the transformation components (refer to enterprises’ ability to combine their existing and newly acquired knowledge) and exploitation components (refer to enterprises’ ability to systematically integrate transformed knowledge into operations to modify, extend, and expand their existing competitiveness) (Zahra and George, 2002; Ali and Park, 2016; Stulova and Rungi, 2017).

Most research analyzed AC from the organizational perspective. However, individual skills and knowledge constitute the AC of enterprises (Santos et al., 2020). Personal observations serve as evidence that is more valid and can better explain the micro fundamentals of organizational change (Lowik et al., 2016; Santos et al., 2020). Cannon et al. (2014) defined AC as “the ability to acquire, assimilate, transform, and exploit knowledge, or to obtain information.” When individuals face and realize the importance of information in the external environment, they absorb and apply that knowledge. This is a demonstration of the personal process.

Based on the above, most research on AC adopted the organizational perspective for discussion, and few studies had examined the issue from a personal perspective. In this study, IAC is the focus of discussion. Following Zahra and George (2002), Cannon et al. (2014), IAC was defined based on the conceptualization of learning and dynamic abilities as the ability of individuals to acquire, assimilate, transform, and exploit knowledge or obtain information. In addition, it is composed of two related capabilities, namely, potential and realized ACs. Potential AC is measured as a formative second-order construct composed of the acquisition and assimilation subdimensions. Realized AC is measured as a formative second-order construct composed of the transformation and exploitation subdimensions.

Applications of the AC concept have become increasingly extensive to achieve the results of organizational goals. It is especially successful when applied to the research field of knowledge creation and innovation results (Mariano and Walter, 2015; Limaj and Bernroider, 2019). The research results of Limaj and Bernroider (2019) indicated that both potential and realized ACs have significantly positive impacts on exploitative innovation, whereas potential AC significantly and positively affects exploratory innovation. However, the relationship between IAC and SEI has not yet been the subject of previous research. For entrepreneurs preparing to launch a business in dynamic environments, the keys to successful entrepreneurship and entrepreneurial abilities include the continuous acquisition, assimilation, transformation, and exploitation of new knowledge flexibly. Therefore, this study proposed that potential and realized ACs have impacts on SEI, leading to the following hypothesis:

Hypothesis 7: Potential absorptive capacity has a significant influence on social entrepreneurial intention.
Hypothesis 8: Realized absorptive capacity has a significant influence on social entrepreneurial intention.

**FIGURE 1:**
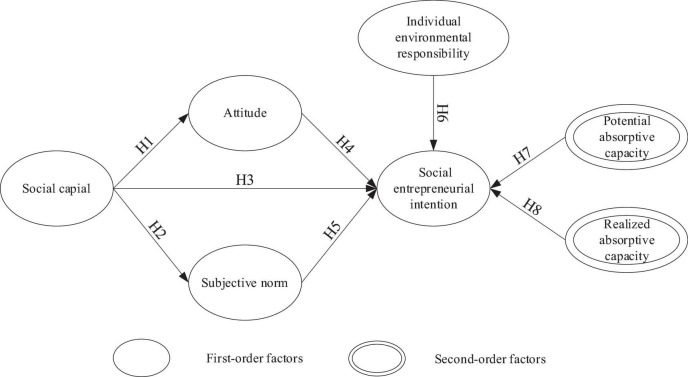
Research model and hypotheses.

This study considered the impacts of SC, attitudes, subjective norms, IER, and potential and realized ACs on SEI. The hypothetical model included: (i) exogenous variables: SC, IER, and potential and realized ACs; and (ii) endogenous variables: attitudes, subjective norms, and SEI (Figure 1). The figure summarizes the hypothetical relationship between the variables and the path model, where the relationships between the constructs (the arrows) represent the proposed research hypotheses.

## Research Methodology

### Instrumentation and Data Collection Tools

The research questionnaire was divided into two sections. The first section covered demographic information such as the respondents’ age, gender, grade of study, and scores for the most recent semester. The second section dealt with the constructs that affected SEI, including the seven variables mentioned in the proposed hypotheses. The constructs were based on the latent variables as measured by multiple-item scales. Most of the items were adapted from previous research, and the measurement items for some variables were expressed with slight adjustments to better match this research.

The main constructs for SC (structural dimension, cognitive dimension, and relational dimension) comprised 13 measurement items, including “I maintain close social relationships with some members of associations in my university,” “In general, members of associations have very good relationships,” “Members of this association always share the same goal of learning from each other,” “Members of this association behave in a consistent manner,” and “Members will always help others if they encounter problems.” The design of the measurement items was adapted from questionnaires developed by earlier scholars (Nahapiet and Ghoshal, 1998; McKeever et al., 2014; Malebana, 2016; Yan and Guan, 2018; Mahfud et al., 2020). The main constructs for TPB included: attitude (4 questions), subjective norms (3 questions), and SEI (5 questions). For example, items include: “I find the idea of being a social entrepreneur attractive,” “If I decided to be a social entrepreneur, my parents would be supportive,” “My family see social entrepreneurship as a logical option,” and “I plan to start a social enterprise upon completion of my studies at the university.” These were adapted from measurement items developed in previous related research (Ajzen, 2015, 1991; Cavazos-Arroyo et al., 2017; Ernst, 2018; Zaremohzzabieh et al., 2019; Sahasranamam and Nandakumar, 2020; Hoa et al., 2021).

The four questions for IER were adapted from the measurement items developed by earlier scholars (Peng and Mao, 2018; Wei et al., 2018; Jia et al., 2021; Yang et al., 2021), items include: “I am willing to increase my purchase of eco-labeled goods to help solve environmental problems,” and “I have a responsibility to help solve environmental problems that society faces.” The main constructs for IAC, comprising potential and realized ACs, included: assimilation (3 questions), acquisition (3 questions), transformation (3 questions), and exploitation (3 questions). These were adapted from previous research (Mariano and Walter, 2015; Limaj and Bernroider, 2019; Audretsch et al., 2021; Miroshnychenko et al., 2021; Qi et al., 2021). For example, items include: “I frequent interactions with some members of associations in my university to acquire new knowledge,” “I have the ability to structure and to use collected knowledge,” “I constantly thinking about how to better exploit knowledge,” “I record and store newly acquired knowledge for future reference,” and “I have the ability to connect existing knowledge with new insights.” The Likert seven-point scale was used for responding to all the measurement items and ranged from “1 = strongly disagree” to “7 = strongly agree.”

The original scale was selected from renowned international journals on SE and entrepreneurship education or management with high reliability and relevance to the current research purpose. This was to ensure accurate evaluation of the proposed research framework and model. Considering that the selected scale was in English, five scholars with extensive research experience in SE and entrepreneurship education were invited to use the back-translation method to ensure that the original meanings were retained and that the scale was suitable for measurement. The reliability and validity of the measurement items in all the measured constructs were ascertained.

A pilot survey was conducted before the formal survey, the feedback data of which were used to analyze the reliability and validity of the research model. Subsequently, detailed modifications were made to some measurement items. Specifically, items with poor reliability and validity were deleted, and the wording of other items was modified. The respondents were 86 undergraduate students from the business administration faculty of a college of science and technology in central Taiwan. Their data were analyzed to understand the Cronbach’s alpha (α) of all constructs. The results show that the Cronbach’s alpha (α) coefficients of all constructs complied with the standard of being higher than 0.7 as recommended by Hair et al. (2010). Thus, all the variables in this study had good reliability and were acceptable.

### Study Participants

The cross-sectional survey methodology was used to collect empirical data from 1,200 undergraduate students from different colleges/universities of business administration in Taiwan (including public universities and science and technology universities). All students who participated in the study were volunteers and were assured that their responses would be anonymous and confidential. In addition, they were allowed to terminate their participation during the process of completing the questionnaire without having to face any consequences. Finally, all participating students had given their written informed consent.

The retrieved questionnaires were screened. Participants with too many answers stating “do not know” or “not applicable” and those with too many missing data due to inconsistent answers were excluded. Eventually, the data of 969 participants were included in the final empirical analysis. The results of the analysis are shown in Table 1. The participants’ average age was 21.09 years (standard deviation = 0.85 years), with males and females comprising 35.8% and 64.2% of the total, respectively. In terms of grade of study, more than half of the participants (56.3%) were in their 3rd year of study.

**TABLE 1:** Survey respondents (*N* = 969).

Demographics/ Level	*N*	Percentage	Demographics/ Level	*N*	Percentage
Gender			Grade		
Male	347	35.8	2nd Grade	379	39.1
Female	622	64.2	3rd Grade	546	56.3
			4th Grade	44	4.5

### Non-response and Common Method Bias

Following the literature, we conducted tests to determine the existence of non-response bias by comparing the early and late response times (Armstrong and Overton, 1977). The results of the chi-square difference test showed that the difference between the two response times was not significant (*p* > 0.0.5). This showed that non-response to the deviation was not the key issue. Following Podsakoff et al.’s (2003) suggestion, Harman’s single-factor test was used with Statistical Package for the Social Sciences (SPSS) 18.0 to check for the potential common method variance (CMV). All measurement items in this study were tested using the exploratory factor analysis of principal component analysis. The results show that the first factor accounted for 36.19% (less than 50%) of the total variance. Therefore, CMV was not the main issue of this study.

## Results

### Data Analysis Method

Structural equation modeling, which was used to analyze this study’s data, consists of the measurement and structural models (Anderson and Gerbing, 1988). The measurement model tests the relationship between the latent variables and corresponding items; the structural model tests the relationship between these latent variables. The SPSS software was used to encode data and evaluate construct reliability. After completing the coding process, the data were stored in a comma-separated values (CSV) file, which was compatible with the Smart partial least squares (Smart) PLS 3.0 software, the analytical tool. The file was then analyzed to evaluate the structural model and test the relationships between the constructs of the hypotheses.

### Measurement Model Estimation

Before verifying the hypotheses, measurement model estimation was made to prove the model’s reliability and validity. Table 2 summarizes the number of items, factor loading, and reliability and validity values of the constructs. The results show that the measurement model’s constructs met all the quality and validity standards. First, the factor loadings of all items were above 0.7 (which exceeded the threshold of 0.6) and reached statistical significance (*p* < 0.05). The standards recommended by Hair et al. (2010) were complied with. The Cronbach’s alpha (α) of all constructs was above 0.8, which meets the standard value of 0.6 recommended by Hair et al. (2010).

**TABLE 2:** Factor loadings, Cronbach’s alpha (α), CR, AVE.

Construct	No. of items	FL	Cronbach’s α	CR	AVE	DV
Social capital (SC)	13	0.776–0.863	0.955	0.960	0.649	2.913
Attitude (ATT)	4	0.800–0.917	0.882	0.919	0.740	1.122
Subjective norm (SN)	3	0.834–0.878	0.826	0.896	0.742	1.577
Individual environmental responsibility (IER)	4	0.740–0.815	0.808	0.870	0.627	3.195
Assimilation (ASS)	3	0.852–0.916	0.867	0.919	0.791	1.414
Acquisition (ACQ)	3	0.755–0.847	0.876	0.910	0.669	1.228
Exploitation (EXP)	3	0.859–0.914	0.869	0.920	0.793	1.362
Transformation (TRA)	3	0.842–0.891	0.822	0.894	0.738	1.268
Social entrepreneurial intention (SEI)	5	0.786–0.894	0.898	0.925	0.710	1.077

*FL, factor loading; CR, composite reliability; AVE, average variance extracted; DV, discriminant validity.*

According to the recommendations of Fornell and Larcker (1981), composite reliability (CR) should meet the fit requirement of 0.6 or higher, and the threshold of the average variance extracted (AVE) was 0.5. The CR values of the research results were in the range of 0.870 to 0.960, and the AVE values ranged from 0.627 to 0.793, which indicates the reliability and convergent validity of all constructs. Finally, the discriminant validity (DV) value should be greater than 1.0, according to the suggestion of previous research (Fornell and Larcker, 1981; Hair et al., 2016). The research results show that the DV values of all constructs met the requirements, indicating satisfactory DV. All constructs of this study were consistent with DV.

### Structural Model Estimation

The structural model was evaluated using three indicators: predictive relevance (Q^2^), the coefficient of determination (R^2^), and hypotheses testing. The R^2^ values of the endogenous constructs show that the proposed model could explain 13.3% of attitudes, 14.8% of subjective norms, and 69.9% of SEI. In addition, the Q^2^ values ranged from 0.097 to 0.490, which were all higher than the 0.000 threshold (Hair et al., 2019). This indicated that the proposed model had sufficient predictive relevance. Based on the above, the model met all the criteria, and the structural model was regarded as good. Finally, the bootstrapping method (5000 resamples) was used to analyze the structural model, and beta coefficient and *t*-statistics were used to test and check the validity of the proposed hypotheses.

**FIGURE 2:**
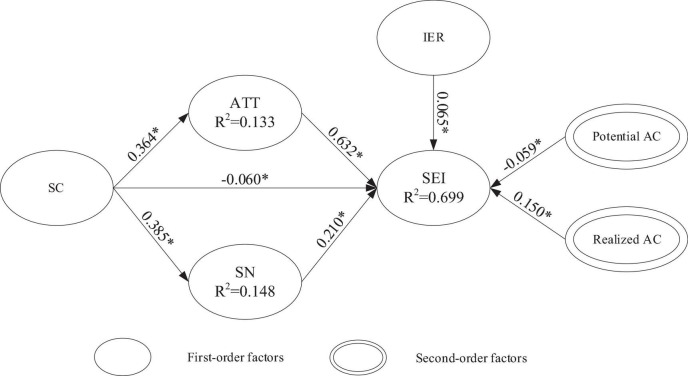
Empirical results of the structural path model. Value on path: standardized coefficients (β), R^2^, Coefficient of determination and *p < 0.05.

The analysis results of the structural model (as shown in Figure 2 and Table 3) indicate that SC had a significantly positive and direct impact on attitudes (H1, β = 0.364, *t* = 10.077) and subjective norms (H2, β = 0.385, *t* = 10.806). However, SC had a significantly negative and direct impact on SEI (H3, β = –0.060, *t* = 2.801). In addition, there were significantly positive and direct impacts on SEI by attitudes (H4, β = 0.632, *t* = 22.781), subjective norms (H5, β = 0.210, *t* = 7.696), IER (H6, β = 0.065, *t* = 3.114), realized AC (H8, β = 0.150, *t* = 4.734). However, potential AC (H7, β = –0.059, *t* = 2.130) had a significantly negative and direct impact on SEI. Therefore, all eight hypotheses proposed in this study were supported. The test results of the structural model’s hypotheses are shown in Figure 2 and Table 3.

**TABLE 3:** Results of path analysis and hypothesis testing.

Hypotheses		Standardized coefficient	*t*-value	Test results
H 1	SC → ATT	0.364*	10.077	Supported
H 2	SC → SN	0.385*	10.806	Supported
H 3	SC → SEI	−0.060*	2.801	Supported
H 4	ATT →SEI	0.632*	22.781	Supported
H 5	SN → SEI	0.210*	7.696	Supported
H 6	IER → SEI	0.065*	3.114	Supported
H 7	Potential AC → SEI	−0.059*	2.130	Supported
H 8	Realized AC → SEI	0.150*	4.734	Supported

*p < 0.05.

### Mediating Effect Analysis

So far, the research model has not examined the importance of attitudes and subjective norms as mediators. Table 3 suggests that attitudes and subjective norms have an intermediary effect. Therefore, mediation analysis was used to test the impact that mediation (attitudes and subjective norms) had on the relationship between the independent and dependent variables. Bootstrap analysis was used to understand the mediating influence of attitudes and subjective norms that SC had on SEI [a bootstrap sample of 5000 was specified, and confidence interval (CI) of 95%]. The mediation path, coefficient, *t*-value, and CI are summarized in Table 4. The bias-corrected test results of the 95% CI show that CI did not contain 0, which confirmed that SC had a significant mediating effect on attitudes and subjective norms. This also meant that SC affected SEI through the mediating role of attitudes and subjective norms. These results show that attitudes and subjective norms made important contributions as mediating variables between SC and SEI.

**TABLE 4:** Results of bootstrap analysis.

Paths	Coefficient	*t*-value	LLCI	ULCI
Social capital → Attitude → Social entrepreneurial intention	0.230*	9.283	0.182	0.280
Social capital → Subjective norm → Social entrepreneurial intention	0.081*	6.605	0.058	0.106

*p < 0.05.

## Discussion

This study empirically tested whether the SEI of undergraduate students in business management was affected by attitudes, subjective norms, SC, IER, and the construct of IAC (i.e., potential absorptive capacity and realized absorptive capacity). In addition, this research advocated for a more detailed understanding of the important roles played by potential and realized ACs in promoting SEI. In terms of academic research, some contributions were made by this research to related fields through connecting SC, IER, IAC, and SEI. Some suggestions pertaining to higher education institutions and practical impacts were also put forward.

Attitudes, subjective norms, SC, IER, and IAC (i.e., potential absorptive capacity and realized absorptive capacity), were identified by this study as essential antecedent factors affecting the SEI of undergraduate students. Specifically, attitudes comprised a critical factor that is extremely related to increasing their EI. This meant that when undergraduate students had a high level of enthusiasm, satisfaction, and positive evaluation of entrepreneurship, their SEI was correspondingly stronger. The second major factor affecting SEI was subjective norms. Our results were consistent with previous studies (Salamzadeh et al., 2013; Malebana, 2016; Cavazos-Arroyo et al., 2017; Ernst, 2018; Zaremohzzabieh et al., 2019) and confirmed our key proposition that attitudes and subjective norms contributed to SEI development. Therefore, vocational educators in colleges should promote positive entrepreneurial attitudes in students and support their willingness to develop new social businesses in their respective fields. However, students’ positive attitudes toward entrepreneurship should also be cultivated in school (for example, students’ perceptions of innovation, achievement, and personal control).

Environmental responsibility is presently regarded as an important research topic given people’s increasing attention to the sustainable development of the environment and is extensively applied. This research attempted to explore the impact of IER on SEI. The results confirmed our key proposition: IER strengthened the SEI of undergraduate students. Because ER is manifested explicitly in individuals’ sense of responsibility to prevent and solve ecological and environmental problems (Peng and Mao, 2018; Jia et al., 2021), they will assume ER when undergraduate students possess these characteristics. Doing so strengthens their environmental awareness and behaviors, thereby affecting their SEI. The results of this research also expanded the application scope of IER in SEI.

Zahra and George (2002) proposed the perspectives of potential and realized ACs, which are widely applied and discussed by subsequent researchers. However, previous studies have rarely addressed the impact of these two variables on SEI. This research regarded the potential and realized ACs of IAC as concepts of a multi-dimensional construct with a higher-order structure. Potential AC includes acquisition and assimilation, and realized AC includes transformation and exploitation. The empirical results confirmed that IAC was an important prerequisite for achieving SEI, although potential AC (i.e., acquisition and assimilation) and realized AC (i.e., transformation and exploitation) were significantly negative and positive impacts on SEI, respectively. This was because potential AC refers to acquiring and assimilating external knowledge and creating knowledge, whereas realized AC is the transformation and exploitation of external knowledge or knowledge utilization.

Although undergraduate students have the ability to obtain information on SE through external resources for analysis, processing, interpretation, and understanding, SE requires not only the absorption of the relevant knowledge but also the presence of social entrepreneurs who share information on their related experiences of success and failure, as well as opportunities for them to practice through entrepreneurial simulation activities. Furthermore, Taiwan’s social environment does not encourage entrepreneurship, and the concept of SE has recently gained attention with few actual cases. If college courses teach SE issues using only the traditional methods, the SEI of undergraduate students would be reduced. Instead, such courses should allow students to systematically use the knowledge that they have acquired and integrate it into the process of SE-related activities. When they understand how knowledge is applied to actual activities, their SEI will strengthen. Vocational educators in colleges can recommend conducting SE courses and hiring social entrepreneurs to teach. Students can also be allowed to experience entrepreneurial activities through entrepreneurial simulation software during the courses.

This study found that SC negatively affected SEI, which was not consistent with previous studies that described the positive effects that SC has on SEI (Malebana, 2016; Ernst, 2018; Zaremohzzabieh et al., 2019). The SC of this study referred to the value that individuals gain through social interactions to develop their social capabilities. It is an actual and potential resource that enterprises can tap through their networks of relationships (Nahapiet and Ghoshal, 1998). For students, their SC is mainly formed through social interactions within the school environment (Mahfud et al., 2020). Their main objects of social interactions are family, friends, teachers, lecturers, and other students, but not social entrepreneurs. Taiwan’s social environment does not support—and even opposes—SE, causing the SEI of undergraduate students to be weak. Therefore, vocational educators in colleges need to develop a teaching method that permits the participation of social entrepreneurs in teaching. This may include having successful social entrepreneurs deliver speeches and conducting courses related to education on SE.

Despite this study’s finding that SC had a negative impact on SEI, our results show that attitudes and subjective norms mediated the impact of SC on SEI. In other words, students’ social support has a great impact and significance on developing their attitudes and subjective norms regarding SE, affecting their SEI. The survey results enhanced our interpretation of the important roles that attitudes and subjective norms play in explaining the impact of SC on the SEI of undergraduate students and further promoted the development of theoretical literature on planned behavior.

## Conclusion

The importance of SEI as the first step toward social entrepreneurial behavior (i.e., social entrepreneurship) has been emphasized. The TPB is the most common theoretical model applied to examine entrepreneurial intention, but it does not present a satisfactory fit for aggregated data. Some researchers, therefore, suggest extending the TPB to improve the quality of outcomes and the explanatory power of behavioral intentions. SC is vital for shaping entrepreneurial intentions. AC studies primarily discuss it from an organizational perspective, whereas few have examined it from the individual’s perspective. The relationship between IAC and SEI has not been the subject of previous research. In addition, IER is a major research issue that has received much attention and application, while its influence on SEI has been rarely studied. In view of the above, this study examined antecedent factors influencing the SEI of business administration students in a university setting using a structural equation model, an extended model with SC, IER, and IAC constructs (i.e., potential absorptive capacity and realized absorptive capacity), and the TPB to fill the gap in this subject, handsomely contributing to the literature. Based on the findings, attitudes, subjective norms, IER, and realized AC positively influenced college students’ SEI, while social capital and potential AC negatively influenced it. Additionally, attitudes and subjective norms played a mediating role in the influence of SC on SEI. These findings are a substantial contribution to the literature, consolidating the theory that SEI is influenced by SC, IER, and IAC. It is recommended that vocational educators develop students’ SEI by instilling entrepreneurial attitudes, IER, SC, and IAC into students through campus and classroom activities.

## Limitations and Future Research

This study has some limitations, even though it covers many concepts and has theoretical and practical implications. First, as with most studies, the results of this study are subject to the cross-sectional study. Future research should use longitudinal studies to test the proposed research model. As new knowledge and experience are accumulated, students’ ability to absorb SE and their understanding of EI will change over time. Future research may adopt a quasi-experimental and related research design, combined with qualitative research, to provide a more in-depth interpretation of the results, and obtain more accurate findings. Second, this study is based on the TPB, and combines several constructs to investigate the factors influencing college students’ SEI. However, in this theory, the antecedent factors that influence behavior do not include the variable of “goal,” and they may be influenced by other variables (e.g., environmental awareness, entrepreneurial education). As such, it is suggested that future research incorporate relevant variables or theories (e.g., Model of Goal-directed behavior, MGB) to devise a more suitable research model and understand the important factors influencing college students’ SEI. Third, the main survey data of this study were from universities in central Taiwan, but people’s SEI vary by region and age group. Further empirical research can include a wider range of age groups, regions, and cultural backgrounds to confirm the validity of the research model proposed in this study.

## Data Availability Statement

The original contributions presented in the study are included in the article/supplementary material, further inquiries can be directed to the corresponding author/s.

## Author Contributions

C-MC collected the data, carried out the data curation and statistical analysis, interpreted the data, obtained the funding, and wrote the original draft. T-KY did the research conceptualization, interpreted the data, supervised the study, and wrote the original draft. Both authors wrote the manuscript together and approved the final manuscript.

## Conflict of Interest

The authors declare that the research was conducted in the absence of any commercial or financial relationships that could be construed as a potential conflict of interest.

## Publisher’s Note

All claims expressed in this article are solely those of the authors and do not necessarily represent those of their affiliated organizations, or those of the publisher, the editors and the reviewers. Any product that may be evaluated in this article, or claim that may be made by its manufacturer, is not guaranteed or endorsed by the publisher.
